# Knowledge, attitudes and perceptions of Nigerian parents towards human papilloma virus (HPV) vaccines

**DOI:** 10.18332/ejm/114886

**Published:** 2020-01-27

**Authors:** Beatrice Ohareri, Abiola Olufunmilayo Adefolaju, Chiemerigo Anne Onyeneho

**Affiliations:** 1 Department of Nursing, Faculty of Clinical Sciences, College of Medicine, University of Ibadan, Ibadan, Nigeria

**Keywords:** knowledge, attitude, HPV vaccines

## Abstract

**INTRODUCTION:**

Human papilloma virus (HPV) is a renowned cause of cervical cancer, which has resulted in high mortality of individuals. Cervical cancer could be reduced by screening and HPV vaccination. This study investigated knowledge, attitudes and perceptions of parents towards HPV vaccines in Ibadan, South-West L.G.A, Oyo State, Nigeria.

**METHODS:**

A cross-sectional descriptive design was used with a multi-stage sampling technique to select 186 parents from Ibadan South-West local government area of Oyo state. A validated structured questionnaire (r=0.78) was used for data collection.

**RESULTS:**

The mean age of the respondents was 30.2 years. The parents had good knowledge of the HPV vaccine (mean=3.12) and most had a high level of knowledge (98.9%). Parents demonstrated negative attitude (mean=2.97) and positive perception to HPV vaccines. Major factors affecting their attitude towards the uptake of HPV vaccines were: finance (86%), level of education (81%), distance to health facilities (83%), inadequate knowledge about the vaccine (89%), fear of promiscuity (82%), and concern about adverse effect (80%).

**CONCLUSIONS:**

Factors responsible for a negative attitude were the high cost, distance from vaccination site, inadequate knowledge, fear of subsequent adolescent promiscuity and concern about adverse effects. In light of the benefits of HPV vaccination, the Nigerian government should make HPV vaccines available, affordable, and accessible to the public.

## INTRODUCTION

Cancer is the second leading cause of death globally and is responsible for an estimated 9.6 million deaths in 2018^1^. Cervical cancer is one of the leading causes of death with about 0.27 million deaths occurring yearly^1^. In Nigeria, cancer of the cervix accounted for 62.3% and 65.7% of histologically confirmed gynaecological cancers, respectively^2,3^. Cervical cancer is a major health problem globally, especially in sub-Saharan Africa including Nigeria^4^.

HPV is the most common viral infection, especially in Nigeria^4^. It is a renowned cause of cervical cancer but also other cancers including of the vulva, anus, vagina, penis, head and neck. Globally, HPV-associated infection incidence is very high, with about 14 million cases in 2008^5,6^.

WHO has endorsed the HPV vaccine as the prime approach for the prevention of cervical cancer, to be administered before first sexual contact. Certain countries have also initiated vaccination against HPV in males, as the vaccines available are found to be effective for the prevention of anal pre-cancers and genital warts in both sexes^7^.

The vaccines are approved for administration to females aged 9–26 years. They were licensed and introduced in Nigeria in 2009, but are being utilized by few privileged populations. Several studies have reported low knowledge of HPV vaccination against the infection and high cost beyond the reach of average Nigerians^8,9^.

Hence, this study was carried out to assess the knowledge, attitudes and perceptions of parents to HPV vaccination in Ibadan, Oyo state, Nigeria.

## METHODS

### Design and setting

A cross-sectional convenience study design was used. The study was carried out on parents in Ibadan South-West local government area of Oyo state. A multi-stage sampling technique was used to select calculated sample size of 186 participants who met the inclusion criteria, using the Kish & Leslie formula.

### Questionnaire

Face and content validity were performed to ascertain the validity of the instrument. A pilot test was carried out, and a test-retest method of reliability was carried out using ten parents twice within a time interval of two weeks. The result was correlated using Pearson moment-correlation coefficient analysis; the resulting reliability coefficient was 0.78, which shows the instrument is reliable. The validated structured questionnaire (r=0.78) was then used for data collection on sociodemographics, knowledge, attitudes and perceptions of HPV vaccine amongst parents and factors affecting attitudes towards the uptake of HPV vaccine (Supplementary file).

Responses of ‘knowledge’ and ‘attitudes’ were analyzed using the mean. The responses were assigned scores as follows: Very True of me=4, True of me=3, Untrue of me=2, Very Untrue of me=1. The mean of each item that assessed knowledge was calculated, and a weighted grand mean was obtained from these means and then used to compare the cut off of 2.5. A weighted grand mean ≥2.5 was defined as a good level of knowledge and vice versa. The level of knowledge was analyzed by assigning scores to the responses as follows: Very True of me=4, True of me=3, Untrue of me=2, Very Untrue of me=1. A total score ≥32 means high level of knowledge and vice versa. Responses of ‘perception’ were also analyzed using the mean. The responses were assigned scores as follows: Strongly agree=4, Agree=3, Disagree=2, and Strongly disagree=1. The mean of each item that assessed perception was calculated and a weighted grand mean was obtained from these means, and then used to compare the cutoff. A weighted grand mean ≥2.5 was noted as a positive perception and vice versa.

Ethical approval was obtained from Oyo State Ministry of Health Ethical Review Committee, for permission to carry out the research in Ibadan South-West Local Government Area. The ethical principles of research were maintained. The consent of the respondents was sought by first explaining the purpose of the research and informing them that their participation was voluntary. They were assured of their anonymity and confidentiality, informed of their freedom to withdraw at any time without any penalty or punishment and then informed consent was obtained.

### Analysis

Data entry, coding and running of data was done using Statistical Package for Social Sciences (SPSS) Version 22. Data were analyzed using descriptive statistics of mean, frequencies and percentages, and inferential statistics.

## RESULTS

The mean age of the respondents was 30.2 years, the majority were females (71.5%), of Yoruba ethnic group (75.3%) and Christians (79.6%) (Table 1). The parents have good knowledge of HPV vaccine with weighted grand mean score of 3.12. Also, most (95%) reported that HPV vaccines are serum and protect against infections caused by HPVs, the vaccination should be given to girls around the ages of nine to thirteen years (83%), and the vaccines provide protection for at least 5 to 10 years (88%). Most (98.9%) had high level of knowledge of HPV vaccine.

**Table 1:** Sociodemographic characteristics of parents, Ibadan, Nigeria (N=186)

*Characteristics*	*Percentage (%)*
**Gender**	
Not specified	5.4
Male	23.1
Female	71.5
**Ethnic groups**	
Yoruba	75.3
Hausa	3.2
Igbo	16.1
Other	5.4
**Religion**	
Christianity	79.6
Islam	16.6
Traditional	1.6
Other	2.2
**Age** (years)	
10–19	10.2
20–29	32.8
30–39	34.4
40–49	12.9
50–59	5.4
60–69	2.2
70–79	2.2
**Marital status**	
Single	29.6
Married	63.4
Divorced	1.1
Widowed	3.8
Separated	0.5
Cohabiting	1.6
**Education**	
Not specified	5.4
Primary	2.7
Secondary	18.8
Tertiary	71.0
No formal	2.2
**Occupation**	
Not specified	1.1
Civil servant	35.5
Trader	35.5
Farmer	4.3
Other	23.7

The majority (72%) reported that they have some reservations for the HPV vaccines, 69% reported that their moral upbringing contradicts HPV vaccines, and their faith contradicts HPV vaccines (61%). Factors affecting their attitude towards the uptake of HPV vaccine were financial barriers (86%), level of education (81%), distance to health facilities (83%), preference for vaccinating girls (79%), lack of awareness that vaccine can be given to males (82%), and inadequate knowledge or information about vaccine (89%) (Table 2).

**Table 2:** Participants response on factors affecting their attitude towards the uptake of human papilloma virus vaccines, Nigeria (N=186)

*No.*	*Statement*	*Strongly agree n (%)*	*Agree n (%)*	*Disagree n (%)*	*Strongly disagree n (%)*
1	Expensive	83 (45)	76 (41)	21 (11)	6 (3)
2	Level of education	66 (36)	85 (45)	26 (14)	9 (5)
3	Distance to vaccination	63 (34)	92 (49)	19 (10)	12 (7)
4	Preference for vaccinating girls than boys	63 (34)	83 (45)	36 (19)	4 (2)
5	The belief that the child is too young to take the vaccine	50 (27)	92 (50)	36 (19)	8 (4)
6	Concern about adverse effects	67 (36)	82 (44)	26 (14)	11 (6)
7	Religion	51 (27)	67 (36)	46 (25)	22 (12)
8	Culture	50 (27)	73 (39)	41 (22)	22 (12)
9	Fear of promiscuity in the vaccinated girl	47 (25)	78 (42)	47 (25	14 (8)
10	Lack of awareness that vaccine can be given to males	50 (27)	103 (55)	30 (16)	3 (2)
11	Inadequate knowledge or information about vaccine	95 (51)	71 (38)	13 (7)	7 (4)
12	Time interval between each dose is too long	26 (14)	89 (48)	58 (31)	13 (7)

Overall, parents had positive perception towards uptake of HPV vaccines with a weighted grand mean of 3.08.

## DISCUSSION

The findings of the study revealed that the parents that responded to our survey in Ibadan, Nigeria, have good knowledge of HPV vaccine. A similar finding reported awareness of HPV vaccine and its relevance to development of cervical carcinoma among parents10. However, a low level of awareness of HPV vaccine was reported by some studies^8,9,11,12^.

Parents demonstrated negative attitude to HPV vaccines. This finding is in line with a study that reported negative attitude to HPV vaccine among parents^13^. However, a study showed a contrary report on attitude towards HPV vaccine among parents^14^. It could be inferred that the negative attitude demonstrated by the respondents towards HPV vaccine uptake based on their religious belief is not unexpected, as Nigeria, with its diverse religions and their practices, has religions that forbid the uptake of drugs, vaccines and even certain health-care procedures.

The parents had positive a perception towards HPV vaccine uptake, as in another study^11^ where favourable opinions were found about sexually transmitted infection (TSI) prevention messages for vaccination, including at young ages, and that sexual health is a topic of conversation between adolescents and healthcare providers. In contrast to this study, a negative perception was reported among female adolescents in selected secondary schools in Ibadan, Nigeria^15^. This presents an opportunity to strengthen vaccination channels, especially for adolescents, in order to prevent HPV.

Factors such as financial barriers, level of education, distance to health facilities, preference for vaccinating girls, misconception about the effect of the vaccines, religion and cultural beliefs, fear of promiscuity, ignorance that males can take the vaccines, and the longevity of dose interval, are the major factors influencing the attitudes of the parents towards uptake of the HPV vaccines. Similar studies reported that ignorance of vaccination availability for boys, safety concerns, belief, physician recommendation, health insurance coverage and cost of vaccination had a significant effect on uptake of vaccination^16,17^. Even though the parents in Ibadan South-West have good knowledge and perception about the HPV vaccine, there were several factors identified that impeded them and their children from taking the HPV vaccine. This might explain why there is an increase in the incidence of cervical cancer in Ibadan^18^. There is, therefore, a need to tackle these factors by means of proper information, subsidization of vaccination, as well as availability of vaccines.

### Strengths and limitations

A strength of the study was the ability of the researchers to get the parents to participate in the study given that in our society the discussion of such sensitive topics is usually difficult because of religion and culture. The limitations of the study are the inability to generalize findings to the overall population of Ibadan, because of the small sample size used (due to financial constraints) and also the cross-sectional design used for the study, which only collects data at a point in time.

## CONCLUSIONS

The study revealed that respondents had good knowledge, high level of knowledge and positive perception of HPV vaccine. Negative attitudes demonstrated by the parents and factors responsible were the expense and distance to health facilities, inadequate knowledge about the vaccine, fear of subsequent adolescent promiscuity and concern about adverse effects. There is a need to make HPV vaccine accessible, available, affordable, and to educate parents more about HPV vaccine and dispel fears about adverse effects through education.

## References

[1] World Health Organization (2019). Cancer: Key facts.

[2] Ijaiya MA, Aboyemi AP, Olatinwo AWO, Buhari MO (2002). Clinicopathological Presentation of primary cervical cancer seen in Ilorin, Nigeria. Nigeria Journal of Surgical Research.

[3] Oguntayo OA, Zayyan M, Kolawole AOD, Adewuyi SA, Ismail H, Koledade K (2011). Cancer of the Cervix in Zaria, Northern Nigeria. Ecancermedicalscience.

[4] Agida ET, Akaba GO, Isah AY, Ekele B (2015). Knowledge and Perception of human papilloma virus vaccine among the antenatal women in Nigeria tertiary hospital. Niger Med J.

[5] Forman D, de Martel C, Lacey CJ (2012). Global burden of human papilloma virus and related diseases. Vaccine.

[6] Park IU, Intracaso C, Dunne EF (2015). Human Papilloma Virus and Genital Warts: A Review of the Evidence for the 2015 Centers for Disease Control and Prevention of Sexually Transmitted Diseases Treatment Guidelines. Clin Infect Dis.

[7] World Health Organization (2013). Comprehensive Cervical Cancer Prevention and control: A Healthier Future for Girls and Women.

[8] Odetola TD, Ekpo K (2012). Nigerian Women’s percepton about human papilloma virus immunizations. J Community Med Health Educ.

[9] Ezenwa BN, Balogun MR, Okafor IP (2013). Mother’s human papilloma virus knowledge and willingness to vaccinate their adolescent daughters in Lagos, Nigeria. Int J Womens Health.

[10] Sherman SM, Nailer E (2018). Attitudes towards and Knowledge about Human Papilloma Virus and the HPV vaccination in parents of teenage boys in the UK Published. PlosOne.

[11] Niccolai LM, Hansen CE, Credle M, Shapiro ED (2016). Parents Recall and reflections on Experiences related to HPV vaccination for their Children. Qual Health Res.

[12] Seven M, Guvenc G, Salin E, Akyuz A (2015). Attitudes to HPV vaccination among parents of children aged 10-13 years. J Pediatr Adolesc Gynecol.

[13] Ratanasiripong NT, Sri-Umporn S, Kathalae D, Hanklang S, Ratanasiripong P (2018). Human Papilloma Virus Vaccination and Factors Related to Intention to Obtain the vaccine among young college women in Thailand. J. Health Res.

[14] Mortensen GL, Adam M, Idtalel L (2015). Parental attitudes towards male HPV: a pan European Cross-sectional Survey. BMC Public Health.

[15] Ndikom CM, Oboh P (2017). Perception, Acceptance and Uptake of Human Papilloma Virus Vaccine among Female Adolescents in Selected Secondary Schools in Ibadan, Nigeria. Afr. J. Biomed. Res.

[16] Newman PA, Logie CH, Lacombe-Duncan A, Baiden P, Tepjan S, Rubincam C (2018). Parents’ Uptake of Human papilloma Virus Vaccines for their Children: a Systematic review and Meta-analysis of Observational Studies. BMJ Open.

[17] Radisic G, Chapman J, Flight I, Wilson C (2017). Factors associated with parents’ attitudes to HPV vaccination of their adolescent sons: A systematic review. Prev med.

[18] Sowunmi A, Alabi A, Fatiregun O, Olatunji T, Okoro US, Durosimi Etti AF (2018). Trend of Cancer Incidence in an Oncology Center in Nigeria. West Afr J Radiol.

