# Supplementary material for: Knowledge, attitudes and perceptions of Nigerian parents towards human papilloma virus (HPV) vaccines
Source: Eur J Midwifery. 2020 Jan 27;4:2. doi: 10.18332/ejm/114886 (PMC7839110; doi:10.18332/ejm/114886)
Supplement: Supplementary file 1 [file EJM-4-2-s1.pdf]

DEPARTMENT OF NURSING

UNIVERSITY OF IBADAN

PARENTAL HUMAN-PAPILLOMA VIRUS VACCINESKNOWLEDGE, ATTITUDE AND PERCEPTION QUESTIONNAIRE (PHPVVKAPQ)

Serial Number ______________

Dear Respondents,

I am undertaking a research project titled **"Knowledge, Attitude and Perception of Parents to Human Papilloma Virus Vaccines in Ibadan South-west, Local Government Area of Oyo State, Nigeria".** There is no right or wrong answers to the questions asked or the statements made, every opinion is welcome, what are anticipated are your truthful and honest responses. Please note that the completion of this questionnaire is entirely voluntary. All information gathered as a result of your participation in this study will be treated with utmost confidentiality and will be used sternly for educational research purposes only.

Thank you.

I have read and understand the consent form and voluntarily agree/disagree to participate in the study by ticking (√) in the appropriate box below:

1. Agree () 2. Disagree ()

________________________ ________________________

Signature Date

SECTION A: SOCIO-DEMOGRAPHIC INFORMATION

Please tick (√) any of the responses that apply to you in the options provided or complete the blank spaces provided as applicable.

1 (a). Age (in years) as at last birthday________________ ☐ ☐

2 Sex/ gender: Male ( ) Female ( )

3 Religion: 1. Christianity ☐ 2. Islam ☐ 3. Traditional ☐ 4. Others (specify)_____

4 Marital Status: 1. Single ☐ 2. Married ☐ 3. Divorce ☐ 4. Widowed ☐
5. Separated ☐ 6. Cohabiting ☐

5 Occupation: 1. Civil servant ☐ Trader 3. Fa☐er 4. Oth☐(specify)_____

6 Ethnic Group: 1. Yoruba 2. Hausa 3. Igbo 4. Others (specify)_________

7 Level of Education: 1. Primary [ ] 2. Secondary [ ] 3. Tertiary [ ] 4. No formal education [ ]

SECTION B: KNOWLEDGE OF HUMAN PAPILLOMA VIRUS VACCINE

Instruction: The table below contains a set of statements to assess your knowledge on Human Papilloma Virus Vaccine. Please read and tick (√) as appropriate. Kindly use the following categories: VT = Very True, T = True,Untrue = U, VU=Very Untrue.

**S/N**	***KNOWLEDGE OF HUMAN PAPILLOMA VIRUS VACCINE***	**Very True**	**True**	**Untrue**	**Very Untrue**
8.	Human Papilloma Virus vaccines are serum that protect against infections caused by Human Papilloma Viruses				
9.	The vaccines protect against either two, four, or nine types of Human Papilloma Virus				
10.	Human Papilloma Virus vaccines can also be given to girls beginning at age 9 years				
11	Human Papilloma Virus vaccines can be given at any age				
12.	All Human Papilloma Virus vaccines does not protect against at least Human Papilloma Virus type 16 and 18 that cause the greatest risk of cervical cancer				
13.	The vaccination should be given to girls around the ages of nine to thirteen years				
14.	The vaccines provide protection for at least 5 to 10 years				
15.	Adolescents who get their first dose at age 15 or older need three doses of vaccine given over 6 months				

16.	Persons who have completed a valid series with any Human Papilloma Virus vaccine do not need any additional doses				
17.	Human Papilloma Virus vaccines are highly effective in preventing infection they target when given before initial exposure to the virus				

SECTION C: ATTITUDE TO HUMAN PAPILLOMA VIRUS VACCINES

Instruction: The table below contains a set of statements to examine your attitude on Human Papilloma Virus Vaccine. Please read and tick (√) as appropriate by using any of these categories: VTM = Very True of Me, TM = True of Me, UM = Untrue of Me, VUM = Very Untrue of Me

**S/N**	***ATTITUDE TO HUMAN PAPILLOMA VIRUS VACCINES ITEMS***	**Very True of me**	**True of me**	**Untrue of me**	**Very Untrue of me**
18.	I have some reservations for the Human Papilloma Virus Vaccines				
19.	My faith contradicts Human Papilloma Virus vaccines.				
20.	In the actual sense, Human Papilloma Virus vaccines is not necessary to be taken				
21.	I am willing to allow my wards take Human Papilloma Virus vaccines				
22.	The vaccines should be encouraged to be taken by all				
23.	My moral upbringing contradicts Human Papilloma Virus vaccines.				

SECTION D: PERCEPTIONS TOWARDS HUMAN PAPILLOMA VIRUS VACCINES

Instruction: The table below contains a set of statements to examine your attitude on Human Papilloma Virus Vaccine. Please read and tick (√) as appropriate by using any of these categories: VTM = Very True of Me, TM = True of Me, UM = Untrue of Me, VUM = Very Untrue of Me

S/N	*ITEMS*	Strongly Agree	Agree	Disagree	Strongly Disagree
24.	The idea of Human Papilloma Virus Vaccine is laudable. Therefore, I support it				
25.	I feel Human Papilloma Virus vaccines as being useful in our present time				
26.	The side effects of Human Papilloma Virus is more than the benefits				
27.	Human Papilloma Virus Vaccines cannot have any side effect				
28.	The healthcare facilities is too far to access Human Papilloma Virus Vaccines				
29.	Human Papilloma Virus Vaccines are not readily available				
30.	Human Papilloma Virus Vaccines are too expensive, so it is better to stay out of it.				
31.	The benefits of Human Papilloma Virus Vaccines outweigh the side effects, so it should be encouraged				

SECTION E: FACTORS INFLUENCING ATTITUDE OF PARENTS TOWARDS THE UPTAKE OF HUMAN PAPILLOMA VIRUS VACCINE

Instruction: The table below contains a set of statements to examine likely factors affecting the uptake of Human Papilloma Virus Vaccine. Please read and tick (√) as appropriate by using any of these categories: SA = Strongly Agree , A= Agree, D = Disagree SD = Strongly Disagree

S/N	Statement	Strongly Agree	Agree	Disagree	Strongly Disagree
32	Finance is a major barrier in the uptake of Human papilloma virus vaccine				
33	Level of Education can affect the use of Human Papilloma virus vaccine				
34	Distance is a barrier to the use of Human papilloma virus vaccine				
35	Preference for vaccinating girls than boys				
36	Belief that child is too young to take the vaccine				
37	Concern about adverse effect				
38	My religion does not permit the uptake of the vaccine				
39	My culture is against the use of Human papilloma virus vaccine				
40	Fear of promiscuity in the vaccinated girl				
41	Lack of awareness that vaccine can be given to males				
42	Inadequate knowledge or information about vaccine				
43	Time interval between each dose is too				

	long				

THANKS FOR YOUR PARTICIPATION

© 2020 Ohaeri B. et al.
